# Staged Endovascular Intervention for Axillary Artery Aneurysm Complicated by Recurrent Upper Limb Arterial Embolism: A Case Report

**DOI:** 10.1002/ccr3.73333

**Published:** 2026-08-24

**Authors:** Xiaolong Li, Yayun Xiao, Yulin Zhang, Liwei Zhang, Qunfeng Liu

**Affiliations:** ^1^ The Department of Thoracic and Cardiovascular Surgery, The First College of Clinical Medical Science China Three Gorges University, Yichang Central People's Hospital Yichang Hubei China; ^2^ Department of Vascular Surgery Zhijiang People's Hospital Zhijiang Hubei China

**Keywords:** axillary artery aneurysm, covered stent graft, endovascular therapy, upper extremity arterial embolism

## Abstract

Axillary artery aneurysm is an uncommon subtype of peripheral arterial aneurysm, and mural thrombus within the aneurysm sac frequently dislodges to trigger recurrent refractory acute upper extremity arterial embolism. This study reports a 57‐year‐old male patient who suffered immediate re‐embolization after isolated percutaneous thrombus aspiration. A staged endovascular strategy consisting of thrombus aspiration, catheter‐directed thrombolysis and covered stent graft implantation was adopted for radical treatment. Perioperative angiography and 2‐month follow‐up computed tomography angiography (CTA) confirmed complete thrombus clearance, aneurysm exclusion and sustained unobstructed distal limb perfusion. This staged minimally invasive protocol addresses both acute distal ischemia and the embolic source at the aneurysm, providing a safe and replicable reference for managing similar complex cases.

## Key Clinical Message

Percutaneous transarterial thrombectomy combined with covered stent graft exclusion via endovascular approach is effective for right axillary artery aneurysm complicated by recurrent upper extremity arterial embolism.

## 1 Introduction

Axillary artery aneurysm (AAA) accounts for less than 1% of all peripheral arterial aneurysms and presents rarely in clinical practice [1]. Mural thrombus attached to the aneurysm wall may shed recurrent emboli, leading to refractory acute upper limb arterial ischemia that poses great therapeutic challenges. Traditional open surgical repair carries substantial operative trauma and high complication risks. Isolated percutaneous thrombus aspiration only alleviates distal vessel occlusion temporarily without eliminating the embolic source, resulting in frequent embolism recurrence. In recent years, endovascular covered stent graft implantation has gained popularity for axillary artery aneurysm management owing to its minimal invasiveness and rapid postoperative recovery. Nevertheless, standardized endovascular workflows for patients presenting with concurrent acute distal arterial embolism have not yet been established. This paper presents a clinical case of true right axillary artery aneurysm with mural thrombus that induced recurrent right upper extremity arterial embolism. A sequential endovascular regimen of “thrombus aspiration + catheter‐directed thrombolysis + covered stent graft exclusion” was successfully implemented to cure the patient, aiming to supply clinical evidence and therapeutic references for analogous cases.

## 2 Case History

**FIGURE 1:**
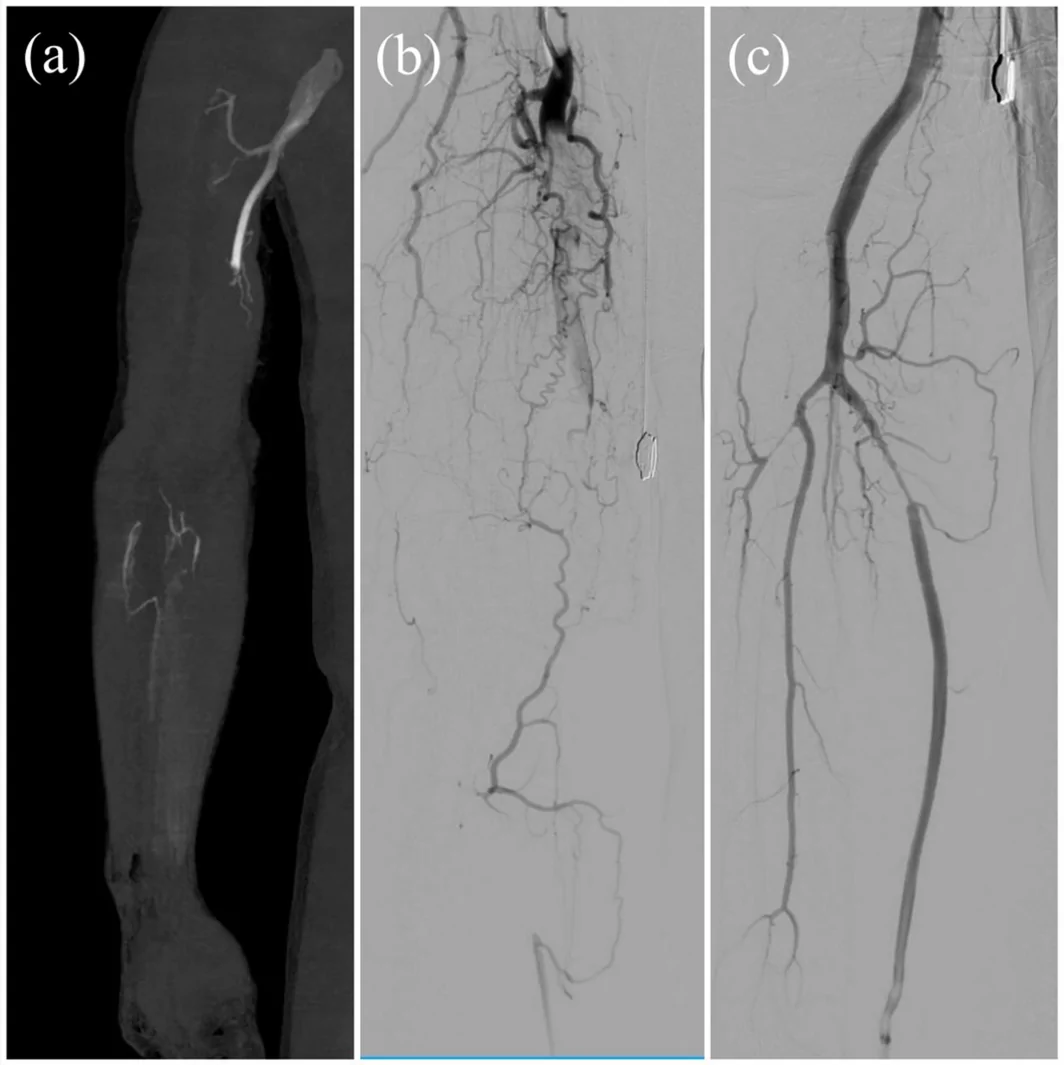
Preoperative CTA and intraoperative angiograms of the first endovascular procedure. (a) Preoperative CTA: Right axillary artery aneurysm with mural thrombus and total occlusion of distal brachial, radial and ulnar arteries; (b) intraoperative angiography before thrombus aspiration: Diffuse thrombotic occlusion of the right upper limb distal arterial tree; (c) post‐aspiration angiography: Restored patency of brachial, radial and ulnar arteries.

A 57‐year‐old male was admitted on January 25, 2026, with a chief complaint of persistent pain and numbness affecting his right upper limb over the preceding 4 days. The patient denied prior hypertension, diabetes mellitus or limb trauma. Physical examination revealed absent radial and ulnar arterial pulses on the right side, decreased skin temperature and cutaneous pallor of the right upper extremity. Preoperative complete right upper limb arterial CTA demonstrated a right axillary artery aneurysm filled with mural thrombus, together with total occlusion of the right brachial, radial, ulnar arteries and their distal branches (Figure 1a).

## 3 Differential Diagnosis

Prior to confirming the definitive diagnosis of recurrent embolism secondary to mural thrombus shedding from axillary artery aneurysm, clinicians must rule out alternative etiologies responsible for upper limb arterial ischemia. Differential diagnoses include cardiogenic thromboembolism, in situ atherosclerotic thrombosis of the subclavian or axillary artery, traumatic pseudoaneurysm, and infectious mycotic aneurysm.

## 4 Investigations and Treatment

### 4.1 Investigations

Besides comprehensive clinical assessment, routine laboratory tests including complete blood count, blood biochemistry, electrocardiogram and cardiac biomarker assay were performed. Electrocardiogram ruled out atrial fibrillation; echocardiography showed normal cardiac function without valvular lesions or intracardiac thrombus. Preoperative blood gas analysis was conducted to screen for concurrent respiratory failure.

### 4.2 Treatment

**FIGURE 2:**
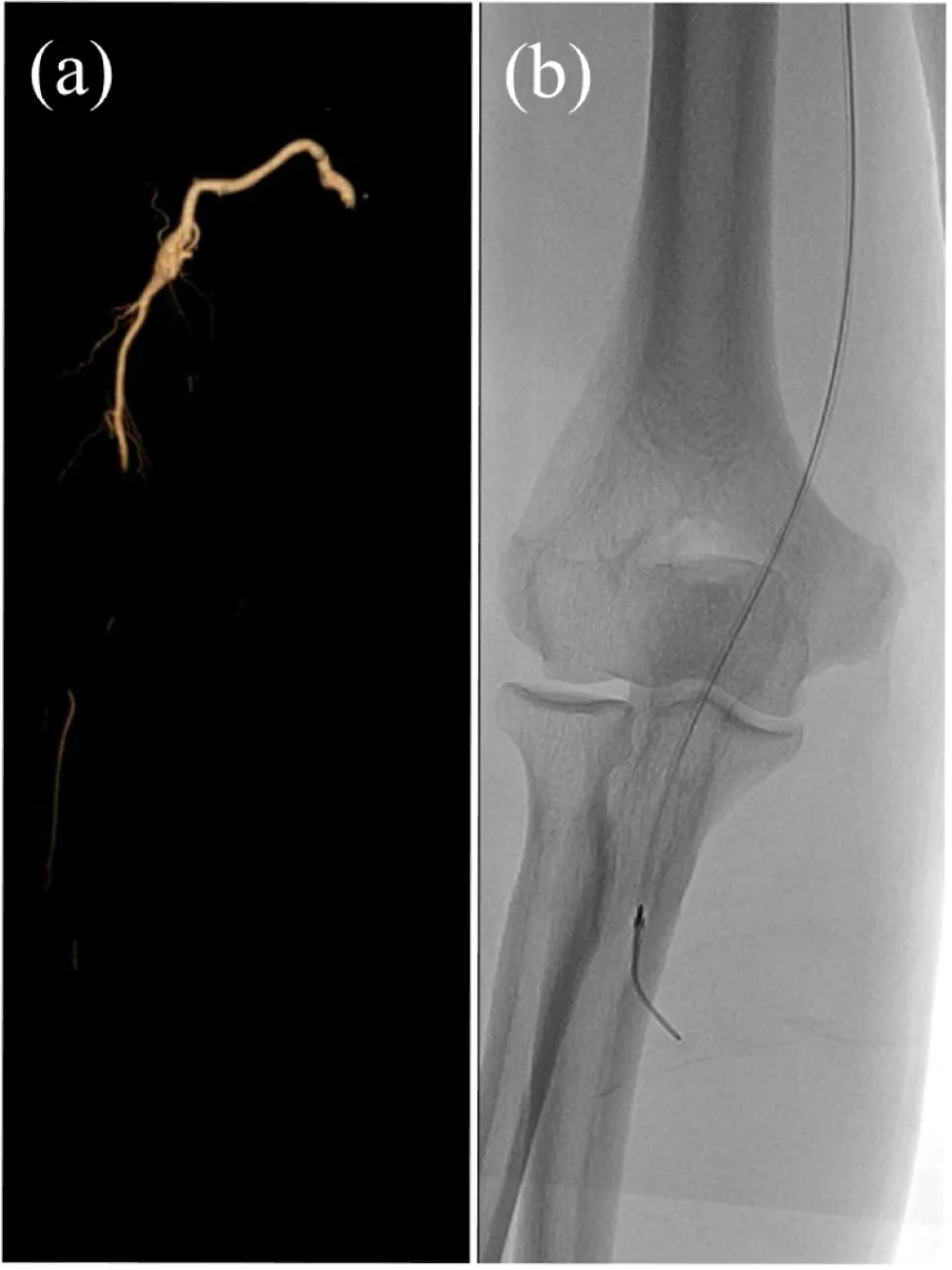
Emergency CTA and intraoperative fluoroscopy of the second intervention. (a) Emergency postoperative CTA after first thrombectomy: Residual mural thrombus within axillary aneurysm and partial re‐occlusion of distal limb arteries; (b) Intraoperative fluoroscopy: Catheter placement within brachial artery for directed thrombolysis.

**FIGURE 3:**
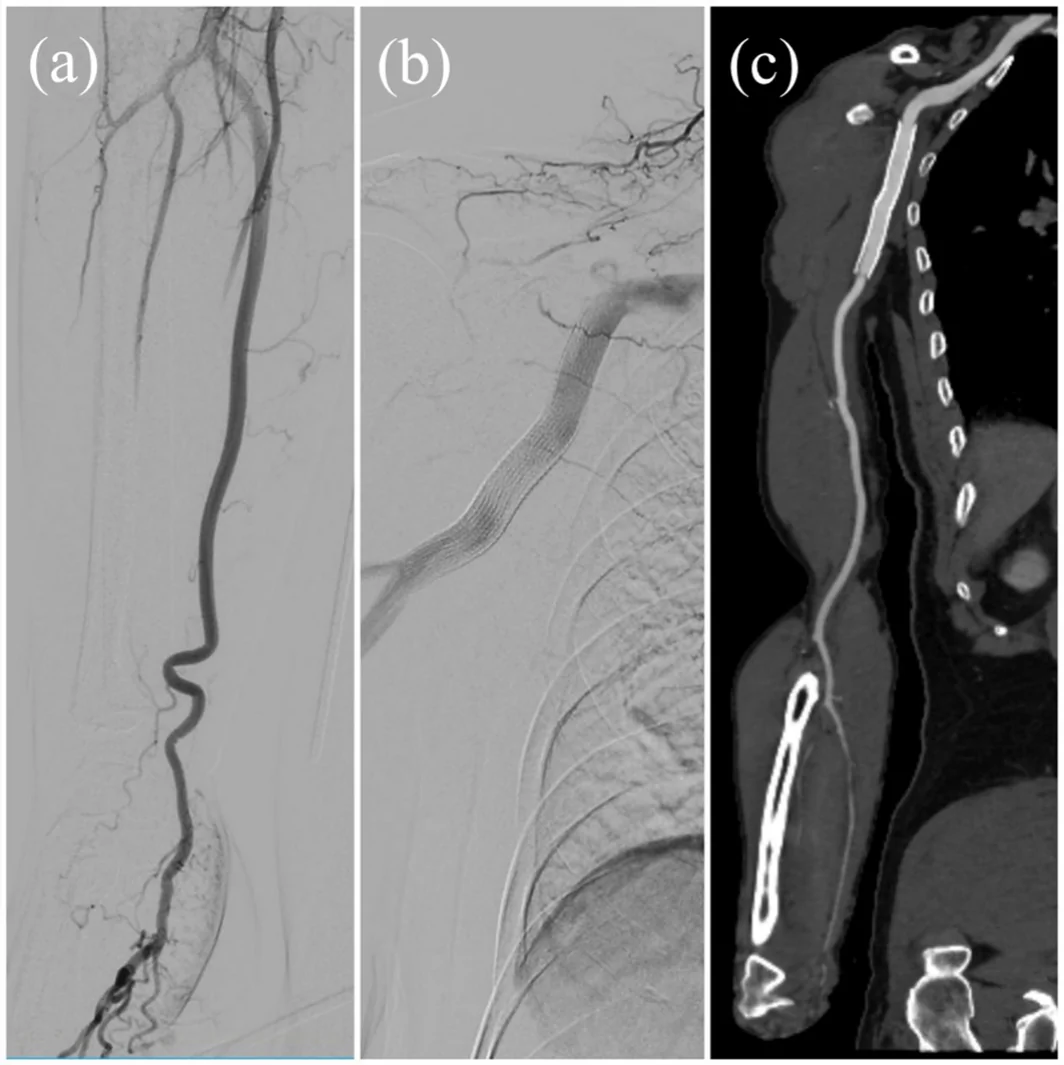
Angiography after thrombolysis, stent deployment and 2‐month follow‐up CTA. (a) Angiography after 3 days of catheter thrombolysis: Complete thrombus clearance with unobstructed distal perfusion; (b) Intraoperative angiography after covered stent graft implantation: A 10 × 80 mm Viabahn stent deployed to exclude axillary artery aneurysm, no endoleak observed; (c) Two‐month follow‐up CTA: Patent axillary stent without migration or endoleak, sustained patency of distal upper limb arteries.

The patient presented with acute right upper limb ischemia secondary to extensive embolism of the brachial, radial and ulnar arteries, so emergency right upper limb angiography and percutaneous arterial thrombus aspiration were scheduled. After completing preoperative preparations, the percutaneous thrombectomy of the right upper limb artery was successfully completed on January 26, 2026. Intraoperative angiography indicates thrombotic occlusion of the right brachial artery, radial artery, ulnar artery, and distal blood vessels (Figure 1b). During the operation, the right upper limb brachial artery and ulnar radial artery were opened, and the radial artery pulsation was good after the operation (Figure 1c). Postoperative anticoagulation and dilation therapy will be administered. At 18:40 on postoperative day 1, the patient developed recurrent right upper limb pain and numbness. Emergency right upper limb arterial CTA revealed residual mural thrombus within the right axillary artery aneurysm, as well as partial re‐occlusion of the brachial, radial and ulnar arteries and their distal segments (Figure 2a). Preoperative plan: Considering that the patient had a thrombus detachment from the wall of the right axillary artery aneurysm, leading to a re‐embolization of the distal end, it is planned to undergo right upper limb arterial angiography, thrombectomy, and catheterization thrombolysis. Emergency surgery was arranged, including right upper limb arterial thrombectomy and catheterization thrombolysis (Figure 2b). After surgery, urokinase is pumped into the thrombolysis catheter daily for treatment, with approximately 500,000 units per day. Three days after thrombolysis, a follow‐up angiography was performed again. During the operation, it was found that the thrombus in the brachial artery and ulnar radial artery had disappeared and the blood flow was unobstructed (Figure 3a). Therefore, a 10 × 80 mm Viabahn covered stent was implanted at the site of the axillary artery aneurysm to isolate it (Figure 3b). After surgery, the patient's ulnar and radial artery pulsation recovered, and the symptoms of pain and numbness in the right upper limb disappeared. After discharge, anticoagulant therapy was continued. Long‐term oral anticoagulation was prescribed upon discharge. Two‐month follow‐up upper limb arterial CTA confirmed patent stent graft without migration or endoleak; the right brachial, radial and ulnar arteries maintained satisfactory patency (Figure 3c).

## 5 Discussion

Axillary aneurysm is a relatively rare but highly threatening peripheral arterial disease, accounting for less than 1% of all peripheral arterial aneurysms [1]. Its etiology is diverse, mainly including atherosclerosis, trauma, infection (such as syphilis, bacterial arteritis), connective tissue disease (such as Behcet's disease), and chronic compression caused by thoracic outlet syndrome [2]. This patient has no history of trauma, infection and connective tissue. It is considered that it is more likely to be an atherosclerotic axillary aneurysm. The distinctive clinical feature of this case lies in massive mural thrombus accumulation within the aneurysmal sac, which acts as a persistent embolic reservoir that repeatedly sheds fragments to trigger distal limb embolism. Clinical manifestations of axillary artery aneurysm vary widely: mass compression of the brachial plexus may induce upper limb pain, numbness or muscle weakness; thromboembolism leads to acute distal limb ischemia; aneurysm rupture may even cause life‐threatening massive hemorrhage. Traditional open surgical repair includes aneurysm resection, mural thrombus evacuation and vascular reconstruction via autologous vein or artificial graft bypass, which delivers definitive lesion eradication but carries substantial surgical trauma. The anatomical complexity of the axillary region—with the aneurysm adjacent to the clavicle and brachial plexus nerve bundles—elevates intraoperative difficulty and complication risks, including permanent nerve injury and persistent lymphatic leakage [3]. With the development of endovascular technology, covered stent implantation has emerged as a superior alternative for axillary aneurysms. Its prominent advantages include minimal surgical trauma, shortened hospital stay and rapid postoperative functional recovery. Mechanistically, covered stents isolate the aneurysmal cavity from systemic circulation, preventing aneurysm rupture, accelerating intrasac thrombus organization, and fundamentally eliminating the embolic source by blocking direct blood contact with the thrombotic aneurysmal wall [4]. In this clinical case, the patient was hospitalized with severe acute upper limb ischemia. Although emergency percutaneous thrombus aspiration rapidly recanalized occluded distal arteries, recurrent embolism occurred within 24 h after the initial intervention. This clinical course vividly demonstrates the persistent thrombogenic risk of axillary artery aneurysm complicated by mural thrombus, and further proves that isolated treatment of distal arterial occlusion without addressing the primary aneurysmal lesion cannot prevent recurrent thrombus shedding and re‐embolization.

We adopted an individualized staged endovascular therapeutic strategy for subsequent intervention. Early re‐embolization after the first procedure guided us to identify the axillary aneurysm as the fundamental embolic source. The second intervention combined percutaneous thrombectomy and catheter‐directed thrombolysis to evacuate residual intra‐sac thrombus and microthrombi in distal runoff vessels, minimizing secondary distal embolism risk and creating a stable outflow tract for subsequent aneurysm exclusion. Three days of local catheter thrombolysis achieved complete clearance of intraluminal thrombus, creating optimal anatomical conditions for covered stent deployment. Subsequent implantation of a Viabahn covered stent simultaneously excluded the aneurysmal cavity and reconstructed physiological arterial blood flow. This sequential “thrombus clearance prior to aneurysm exclusion” treatment pathway reflects a balanced clinical strategy that prioritizes emergency limb salvage while targeting the underlying pathological source for radical cure. Compared with one‐stop simultaneous thrombectomy and stent implantation, the staged protocol avoids squeezing residual unstable thrombus toward distal vessels during stent deployment, which greatly reduces the risk of fatal distal microembolism in patients with heavy aneurysmal thrombus burden. For patients presenting with axillary artery aneurysm combined with acute recurrent upper limb arterial embolism, clinicians should avoid merely relieving distal ischemic symptoms without eliminating the primary embolic source. Endovascular covered stent graft implantation provides a minimally invasive, safe and effective radical therapeutic option for such rare lesions. Individualized staged comprehensive endovascular protocols can achieve complete exclusion of the aneurysm while preserving durable distal limb perfusion, which can be generalized as a standardized management scheme for analogous complex peripheral arterial lesions.

## 6 Conclusion

Although axillary artery aneurysm remains a rare vascular disease, its risk of recurrent distal embolism cannot be overlooked. Endovascular covered stent graft implantation provides a minimally invasive and effective therapeutic option for this entity. For patients presenting with acute distal arterial embolism, individualized staged comprehensive endovascular protocols enable safe radical resection of the primary aneurysm while preserving sufficient limb blood perfusion.

## Author Contributions

**Liwei Zhang:** conceptualization. **Qunfeng Liu:** conceptualization, writing – original draft. **Yulin Zhang:** data curation, conceptualization. **Xiaolong Li:** conceptualization, writing – review and editing, writing – original draft. **Yayun Xiao:** conceptualization.

## Funding

The authors have nothing to report.

## Consent

Written informed consent was obtained from the patient for publication of this case report and all associated imaging data. All identifiable personal information has been removed to protect patient privacy.

## Conflicts of Interest

The authors declare no conflicts of interest.

## Data Availability

The data that support the findings of this study are available on request from the corresponding author. The data are not publicly available due to privacy or ethical restrictions.
